# Effects of grazing exclusion on soil microbial diversity and its functionality in grasslands: a meta-analysis

**DOI:** 10.3389/fpls.2024.1366821

**Published:** 2024-03-19

**Authors:** Xiangyang Shu, Qinxin Ye, Han Huang, Longlong Xia, Hao Tang, Xingyi Liu, Jianwei Wu, Yiding Li, Yanyan Zhang, Liangji Deng, Weijia Liu

**Affiliations:** ^1^ Key Laboratory of Land Resources Evaluation and Monitoring in Southwest, Ministry of Education, Sichuan Normal University, Chengdu, China; ^2^ Institute of Agricultural Bioenvironment and Energy, Chengdu Academy of Agriculture and Forestry Sciences, Chengdu, China; ^3^ College of Economics and Management, Xinjiang Agricultural University, Urumqi, China; ^4^ State Key Laboratory of Soil and Sustainable Agriculture, Institute of Soil Science, Chinese Academy of Sciences, Nanjing, China; ^5^ College of Resources, Sichuan Agricultural University, Chengdu, China

**Keywords:** grazing exclusion, microbial diversity, ecosystem function, meta-analysis, grassland

## Abstract

Grazing exclusion (GE) is considered an effective strategy for restoring the degradation of overgrazed grasslands on the global scale. Soil microbial diversity plays a crucial role in supporting multiple ecosystem functions (multifunctionality) in grassland ecosystems. However, the impact of grazing exclusion on soil microbial diversity remains uncertain. Here, we conducted a meta-analysis using a dataset comprising 246 paired observations from 46 peer-reviewed papers to estimate how GE affects microbial diversity and how these effects vary with climatic regions, grassland types, and GE duration ranging from 1 to 64 years. Meanwhile, we explored the relationship between microbial diversity and its functionality under grazing exclusion. Overall, grazing exclusion significantly increased microbial Shannon (1.9%) and microbial richness (4.9%) compared to grazing group. For microbial groups, GE significantly increased fungal richness (8.6%) and bacterial richness (5.3%), but decreased specific microbial richness (-11.9%). The responses of microbial Shannon to GE varied among climatic regions, grassland types, and GE duration. Specifically, GE increased microbial diversity in in arid, semi-arid, and dry sub-humid regions, but decreased it in humid regions. Moreover, GE significantly increased microbial Shannon in semidesert grasslands (5.9%) and alpine grasslands (3.0%), but not in temperate grasslands. Long-term (>20 year) GE had greater effects on microbial diversity (8.0% for Shannon and 6.7% for richness) compared to short-term (<10 year) GE (-0.8% and 2.4%). Furthermore, grazing exclusion significantly increased multifunctionality, and both microbial and plant Shannon positively correlated with multifunctionality. Overall, our findings emphasize the importance of considering climate, GE duration, and grassland type for biodiversity conservation and sustainable grassland ecosystem functions.

## 1 Introduction

The ongoing climate and biodiversity crises pose imminent threats to both ecosystems and human society (Pörtner et al., 2023). Occupying approximately 40.5% of the ice-free land area and harboring most of Earth’s terrestrial biodiversity (Petermann and Buzhdygan, 2021), grasslands offer a wide range of ecosystem services and functions, including biodiversity conservation, soil formation and retention, water regulation, pest control, and cultural services delivery (Bengtsson et al., 2019; Sollenberger et al., 2019; Wang et al., 2022). Meanwhile, grassland ecosystems account for ~34% of the terrestrial carbon stock, playing a crucial role in mitigating climate change (Bai and Cotrufo, 2022; Liu et al., 2023). However, approximately 49% of global grasslands are estimated to be severely degraded, leading to loss of biodiversity and multiple ecosystem functions, primarily due to factors such as overgrazing (Bardgett et al., 2021; Buisson et al., 2022; Sun et al., 2022). Grazing exclusion (GE) programs aim to address the adverse effects of overgrazing and facilitate grassland restoration by excluding livestock grazing in targeted regions (Sun et al., 2021). Previous studies have demonstrated that GE can improve soil quality, increase plant biomass, and facilitate carbon sequestration in grassland ecosystems (Hu et al., 2016; Zhan et al., 2022; Qu et al., 2024). However, these effects are largely dependent on the environmental context.

Soil microbial communities, serving as a cornerstone of biodiversity, play a crucial role in multiple ecosystem functions (multifunctionality, EMF), such as plant growth, soil fertility, hydrology, greenhouse emission, and soil carbon storage and turnover (Delgado-Baquerizo et al., 2016; Coban et al., 2022; Jansson, 2023; Wu et al., 2024). The exclusion of grazing livestock can impact soil microbial diversity through changes in plant species or biomass, or soil pH, and the availability of carbon and/or nutrients (Zhang et al., 2018; Wang et al., 2021; Zhou et al., 2023). To date, the effect of grazing exclusion on microbial diversity remains fragmented and in need of synthesis. Some studies have observed that grazing exclusion can significantly increase microbial diversity (Wang et al., 2019; Zhang et al., 2019; Su et al., 2023), while others have found negligible or negative effects on soil microbial diversity (Li et al., 2022; Fan et al., 2023; Guo et al., 2023). Furthermore, the direction and magnitude of response of microbial diversity to GE are largely determined by the duration of grazing exclusion and vary with microbial group, grassland type, soil depth, and climate conditions (Aldezabal et al., 2015; Yao et al., 2018; Chen et al., 2020; Peng et al., 2021; Wu et al., 2022). Gaining insights into the patterns and controlling factors of grazing exclusion on soil microbial diversity is crucial for biodiversity preservation and resilience against grassland degradation.

Microbial diversity and ecosystem multifunctionality are often positively linked across space and time in natural ecosystems (Jing et al., 2015; Delgado-Baquerizo et al., 2017; Yuan et al., 2020). However, growing evidence suggests that this relationship depends on environmental context and varies along gradients (Hu et al., 2021; Jia et al., 2022; Berlinches De Gea et al., 2023). To date, it remains unclear if positive microbial diversity-multifunctionality relationships persist after the exclusion of grazing livestock. This knowledge gap hinders the identification of potential microbial diversity-function trade-offs during grazing exclusion. Moreover, most studies on biodiversity and multifunctionality changes have focused on local or regional scales (Liu et al., 2022; Zhang et al., 2023; Zheng et al., 2023), with few assessing microbial diversity and function changes during grazing exclusion across global scales, varying climates, and grassland types. Therefore, assessing relationships between microbial diversity and ecosystem functionality under grazing exclusion could inform sustainable management strategies in managed ecosystems and support global grassland restoration initiatives.

Here, we synthesized available data to estimate the influence of grazing exclusion on soil microbial diversity and its functionality. Our meta-analysis included important information on soil microbial diversity and multiple ecosystem functions (i.e., plant biomass, soil fertility, microbial biomass, and SOM decomposition) in global grassland ecosystems. The aims of our study are addressed through the following key questions: (1) Quantify the influence of GE on microbial diversity and its functionality; (2) Estimate the response ratio of various contexts such as various grassland types, climatic regions, soil depths, and grazing exclusion duration on GE’s effect; 3) Determine the correlation between microbial diversity and its functionality under grazing exclusion. We hypothesized that: (1) long-term GE could show a positive effect on soil microbial diversity; (2) Influences of GE on soil microbial diversity are dependent on microbial groups, grassland types, soil depths, and GE duration because grazing exclusion often has different responses to each of them or their combination; (3) Soil microbial diversity show a positive relationship between ecosystem multifunctionality under grazing exclusion.

## 2 Materials and methods

### 2.1 Data collection

**Figure 1:**
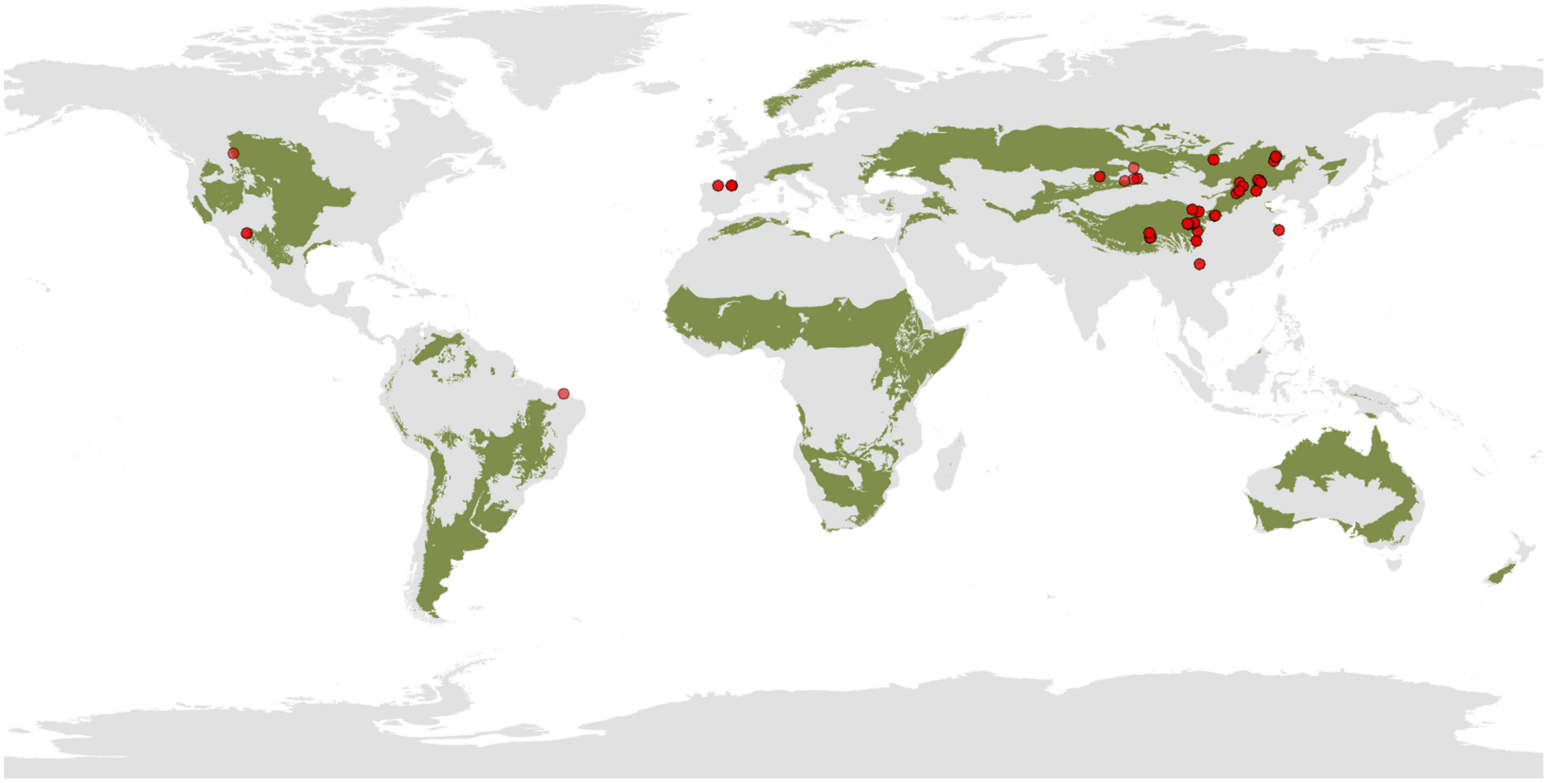
Distribution of study site in the meta-analysis. The green area indicates the distribution of grasslands.

We conducted a comprehensive literature survey using Google Scholar, Web of Science, and China National Knowledge Infrastructure (CNKI) databases up to March 2023, without limiting the publication year. The literature was explored using the following keywords: (grazing exclusion OR fenc* OR enclosure OR grazing prohibition) AND (microb* OR bacteria* OR fung* OR microbial diversity OR OTU OR ASV OR DNA) AND (soil). Additionally, we consulted the reference lists of selected papers to ensure a thorough exploration of relevant studies within the search topics. The selection of eligible studies was based on the following criteria: (1) field-manipulated experiments must encompass both experimental treatments (grazing exclusion) and controls (grazing group); (2) microbial Shannon or microbial richness must be documented; 3) if a single paper contained multiple independent experiments, each experiment was considered as a distinct study and included in our dataset as an independent observation; and 4) the means, the number of replicates, and standard error or standard deviation were provided. If standard error was reported in the literature, we converted it to standard deviation by multiplying by the square root of the number of replicates. After filtering the literature, we identified 48 peer-reviewed publications with 246 paired observations that matched the requirements of our meta-analysis (**Supplementary Figure S1**). Additionally, our dataset collected site locations and climate information. In cases where the original paper did not provide local climate information, mean annual temperature (MAT) and mean annual precipitation (MAP) were sourced from the WorldClim dataset using longitude and latitude coordinates. The MAT of the study sites in our meta-analysis varied between -3.9 to 21.6 °C, and MAP varied between 65.9 to 1814 mm. The duration of GE ranged from 1 to 64 years. The geographical distribution of the sites in our meta-analysis was illustrated in **Figure 1**.

### 2.2 Assessment of ecosystem multifunctionality index

Given that microbial communities drive ecosystem functions, the following variables involved in plant, soil fertility, and SOM decomposition were also collected. (1) For plant communities, we recorded the plant Shannon index and belowground biomass (BGB). (2) For soil variables, we collected soil pH, moisture, soil organic carbon (SOC), soil total nitrogen (TN)/phosphorus (TP), soil available nitrogen (AN)/phosphorus (AP), microbial biomass carbon (MBC)/nitrogen (MBN), β-glucosidase (BG), β-1,4,-N-acetyl-glucosaminnidase (NAG), and phosphatase (Pho). The EMF index was calculated as the average value of all the above ecosystem functions, excluding plant Shannon index, soil pH, and moisture.

### 2.3 Data analysis

The impacts of GE on soil microbial diversity and its functionality were quantified. The natural logarithm of the response ratio (RR) was used to assess the impacts of GE on target variables as the following Equation 1:


(1) $$\text{RR}={\text{ln X}}_{\text{GE}}-{\text{ln X}}_{\text{GG}}$$

where X_GE_ and X_GG_ represent the mean value of the exclusion of grazing livestock and grazing group, respectively. We used Δ pH (i.e., pH (grazing exclusion) – pH (grazing group)) to indicate the influences of grazing exclusion on soil pH (Zhou et al., 2020). Density distributions of the response ratios of target variables are shown in **Supplementary Figure S2**.

The variance in the observations (ν) was calculated using the following Equation 2:


(2) $$v=\frac{S_{GE}^{2}}{N_{GE}X_{GE}^{2}}+\frac{S_{GG}^{2}}{N_{GG}X_{GG}^{2}}$$

where N_GE_ and N_GG_ represent the sample size of grazing exclusion and grazing group, respectively; S_GE_ and S_GG_ denote the standard deviations (SD) of the exclusion of grazing livestock and grazing group, respectively.

The effect size was back-transformed and expressed as percentage change (%) according to the following Equation 3:


(3) $$Percentage\;change\;\left(\%\right)=\left(e^{lnRR}-1\right)\times 100$$

If the 95% confidence intervals overlapped with zero, it denoted that grazing exclusion had no significant effects on the target variables; conversely, if there was no overlap with zero, this denoted a significant effect.

In order to clarify the significant predictors of GE on soil microbial diversity and its functionality, we extracted information on climate regions, grassland types, grazing exclusion duration and soil depths. Climatic regions were determined by aridity index and categorized arid (17 observations), semi-arid (179 observations), dry sub-humid (18 observations), and humid regions (32 observations) (Zomer et al., 2022). Grassland type was grouped into alpine grasslands (81 observations), temperate grasslands (96 observations), semidesert grasslands (51 observations), and tropical grasslands (2 observations) (Dixon et al., 2014). The duration of grazing exclusion was categorized into short- (<10 years), medium- (10~20 years), and long-term (>20 years). Soil depth was divided into two categories (topsoil (0~20 cm) and subsoil (>20 cm)). Specifically, microbial group was grouped by bacteria, fungi, and specific microbes (diazotroph, 1 observation; denitrifier, 3 observations; nitrifier 2 observations, and arbuscular mycorrhizal fungi, 15 observations).

### 2.4 Publication bias

Publication bias analysis is a crucial component of meta-analytical studies, aimed at examining the potential distortion in the literature due to the selective publication of studies. Understanding and addressing publication bias is essential for ensuring the validity and reliability of meta-analytical findings. In this study, we employed Egger’s tests, a widely used method for detecting publication bias in meta-analyses. Our results showed that there was no potential publication bias in our data (**Supplementary Table S1**).

### 2.5 Statistical analysis

Before statistical analyses, all data were tested for normality. The overall effect of grazing exclusion on target variables was estimated by random-effects model. The probability density distributions for all target variables were fitted using the “ggridges” function within the R package. We analyzed the effects of grazing exclusion among different subgrouping categories, and calculated the between-group heterogeneity (Q_b_). Groups with small sample size (< 3) were removed from these analyses. Additionally, we checked the linear regression relationship between microbial diversity and grazing exclusion duration, mean annual temperature (MAT), mean annual precipitation (MAP), and ecosystem functions. All statistical analyses and graph drawing were performed using R 4.2.3.

## 3 Results

### 3.1 Effects of GE on soil microbial diversity

**Figure 2:**
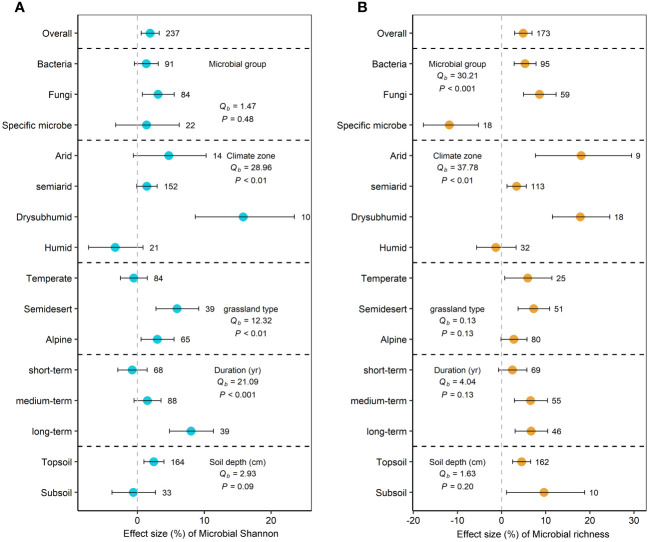
Effects of grazing exclusion on microbial Shannon **(A)** and microbial richness **(B)** across climatic regions, grassland types, grazing exclusion duration, and soil depths. Q_b_ indicate between-group heterogeneity for microbial variables. Means and 95% confidence intervals (CI) are given. The numbers above or below the 95%CI indicate the sample size.

Compared to grazing group, GE significantly increased microbial Shannon and microbial richness by 1.9% (95% CI=0.6 to 3.2%, *P*<0.05) and 4.9% (95% CI=2.9 to 6.9%, *P*<0.05), respectively (**Figure 2**). For microbial groups, GE only significantly increased fungal Shannon (3.1%) (*P*<0.05) (**Figure 2A**). Moreover, GE significantly increased bacterial richness and fungal richness by 1.3% and 3.1% (*P*<0.05), but significantly decreased the richness of specific microbes by 11.9% (**Figure 2B**).

### 3.2 Drivers of soil microbial diversity to grazing exclusion

**Figure 3:**
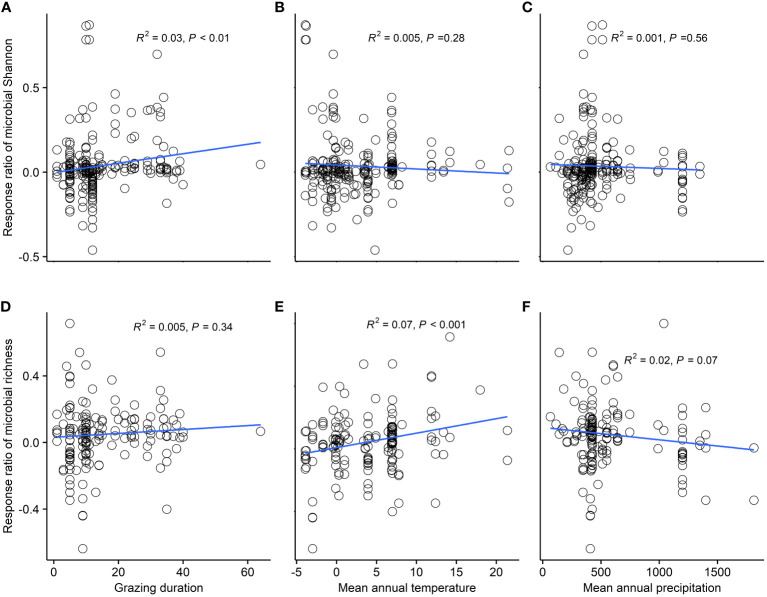
Relationship of microbial diversity and grazing exclusion duration. **(A)** Linear relationships between grazing exclusion duration and response ratio of microbial Shannon **(A)** and richness index **(D)**. Relationship of microbial diversity and grazing exclusion duration and climate factors. **(B)** Linear relationships between mean annual temperature and response ratio of microbial Shannon **(B)** and richness index **(E)**. **(C)** Linear relationships between mean annual precipitation and response ratio of microbial Shannon **(C)** and richness index **(F)**.

Climatic region, grassland type, and GE duration were significant predictors of microbial Shannon response (*P*<0.05). Microbial Shannon index significantly increased in dry subhumid regions (15.8%) and semidesert grasslands (5.9%) and alpine grasslands (3.0%) (*P*<0.05) (**Figure 2A**). Microbial richness index increased in arid (18.0%), semiarid (3.4%), and dry sub-humid regions (13.8%) (*P*<0.05) (**Figure 2B**). Long-term (>20 year) GE significantly increased microbial Shannon by 8.0% (*P*<0.05) (**Figure 2A**). We explored the relationship between microbial diversity and GE duration and between climate factors (**Figures 3A–F**). Response ratios of microbial Shannon were related to GE duration (**Figure 3A**). Besides, response ratios of microbial richness were related to MAT (**Figure 3E**).

### 3.3 Response of individual ecosystem attributes to grazing exclusion and its relationship with microbial diversity

**Figure 4:**
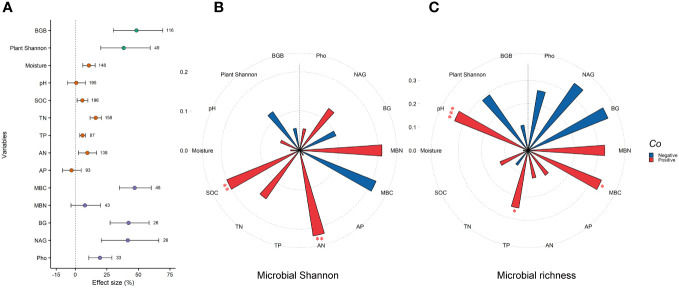
Grazing exclusion affect plant, soil and microbial variables associated with microbial diversity. Response ratio of plant (e.g., plant Shannon and BGB), soil (e.g., soil pH, moisture, SOC, TN, TP, AN, and AP), and microbial variables (MBC, MBN, BG, NAG and Pho) to grazing exclusion **(A)**. Relationship between response ratio of soil microbial Shannon index and plant, soil, and microbial related variables. **(B)** Relationship between response ratio soil microbial richness index and plant, soil, and microbial related variables. **(C)** BGB, belowground biomass; SOC, soil organic carbon; TN, total nitrogen; TP, total phosphorus; AN, available nitrogen; AP, available phosphorus; MBC, microbial biomass carbon; MBN, microbial biomass nitrogen; BG, β-glucosidase; NAG, β-1,4,-N-acetyl-glucosaminnidase; Pho, phosphatase. * *P*<0.05, ** *P*< 0.01, *** P < 0.001.

GE significantly increased plant Shannon and belowground biomass by 38.4% and 48.5%, respectively (*P*<0.05). For soil variables, GE significantly increased soil moisture (10.8%), SOC (5.6%), TN (16.2%), TP (5.8%), and AN (9.6%). Moreover, grazing exclusion significantly increased MBC, BG, NAG, and Pho by 47.1%, 42.2%, 41.6%, and 19.5%, respectively (**Figure 4A**). Response ratios of microbial Shannon were positively related to SOC and AN (*P*<0.01) (**Figure 4B**). Response ratios of microbial richness were positively related to pH, TP, and MBC (*P*<0.05) (**Figure 4C**).

### 3.4 Response of EMF index to grazing exclusion and its relationship with biodiversity

**Figure 5:**
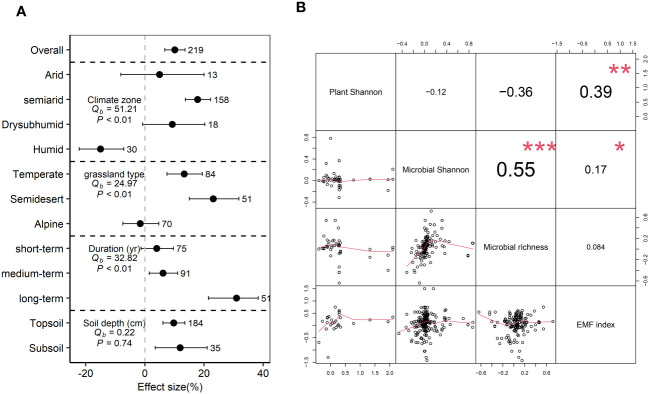
Grazing exclusion affect ecosystem multifunctionality index associated with biological diversity. Response ratio of ecosystem multifunctionality (EMF) index to grazing exclusion across different climatic region, grassland type, grazing duration, and soil depth **(A)**. Q_b_ indicate between-group heterogeneity for microbial variables. Relationship between response ratio soil microbial diversity, plant Shannon index and EMF index **(B)**. * *P*<0.05, ** *P*< 0.01, *** *P*< 0.001.

Grazing exclusion significantly increased EMF index by 10.1% (*P*<0.05) (**Figure 5A**). The EMF index significantly increased in semi-arid regions (17.8%), but decreased in humid regions (-15.0%) (*P*<0.05). Grazing exclusion significantly increased the EMF index in temperate (13.3%) and semidesert (23.1%) grasslands (*P*<0.05) (**Figure 5A**). A positive relationship was observed between the EMF index and microbial Shannon and plant Shannon (*P*<0.05) (**Figure 5B**).

## 4 Discussion

### 4.1 Positive effects of grazing exclusion on microbial alpha diversity

Restoring degraded grasslands presents a complex, long-term challenge (Moreno-Mateos et al., 2020), with grazing exclusion (GE) considered an effective practice for combating degradation and enhancing the recovery of ecosystem services. Numerous studies have investigated GE’ s influence on vegetation community dynamics (e.g., biomass, diversity, and coverage) and soil variables (e.g., pH, moisture, SOC and nutrient availability) (Yu et al., 2021; Zheng et al., 2023). However, understanding of how GE affects soil microbial diversity remains limited.

Pooling data across microbial groups, climatic regions, grassland types, and experimental conditions, our results found GE increased microbial Shannon and richness indices compared to grazing group. This supported our hypothesis 1 and indicated GE is feasible for the preserving microbial diversity in grasslands. This increase in microbial diversity likely stems from GE-driven vegetation and soil improvement, resulting in enhanced microbial diversity (Cheng et al., 2016; Wang et al., 2021). For instance, GE-induced improvement in plant diversity could enhance exudate release and nutrients availability, thereby increasing soil microbial diversity. Additionally, strengthened feedbacks between soil microorganisms and plant communities under GE could contribute to this increase. Despite both plant and soil variables under GE influenced microbial diversity, soil impacts were more pronounced compared to vegetation (**Figure 4**). This suggests that soil conditions may significantly enhance microbial diversity more than vegetation.

### 4.2 Factors regulate the response of microbial alpha diversity to grazing exclusion

For different microbial groups, we observed that fungal diversity was more sensitive to GE than bacterial diversity, consistent with previous studies (Chen et al., 2020; Ju et al., 2023; Wang et al., 2023). Soil fungal communities appear to be more closely associated to grazing exclusion-induced changes in plants than soil bacterial communities, potentially through various pathways such as nutrient absorption and shifts in plant fitness. Moreover, we found that fungal Shannon was positively related to AN, suggesting the increase in fungal Shannon index is related to enhanced soil nitrogen availability induced by GE (**Supplementary Table S1**) (Wang et al., 2020). Importantly, we found GE reduced the richness of some special microorganisms, particularly chitin-degrading bacteria, nitrifiers and denitrifies. This reduction may explain the increased soil nitrogen content under GE, as these microorganisms drive the nitrogen decomposition and loss (Song et al., 2019; Hui et al., 2020; McClure et al., 2022). Additional field studies are warranted to clarify the effects and mechanisms of GE on specific microbes in grassland ecosystems.

The response of microbial diversity to GE is bidirectional across climatic regions: microbial diversity increased in in arid, semi-arid, and dry sub-humid regions, whereas it decreased in humid regions. In general, arid, semi-arid, and dry sub-humid grasslands have lower soil microbial activity and plant productivity compared to humid grasslands. Therefore, by removing grazing pressure, soil microbial communities may have an opportunity to thrive in these productive environments. Possible reasons for the decrease in microbial diversity in humid regions include: (a) GE can decrease soil pH in humid regions (**Supplementary Figure S3**) (Hong et al., 2021), which is unfavorable for microbial growth and thus reduces microbial diversity. (b) Decreased soil bulk density and increased porosity from GE may enhance soil permeability and leaching (Chen et al., 2023), leading to carbon and nutrient loss in humid grasslands. This is supported by our findings of decreased total nitrogen (TN), available nitrogen (AN), and available phosphorus (AP) under GE in humid regions (**Supplementary Figure S3**).

Different grassland types exhibit regional dependence, characterized by varying climatic patterns, terrain, soil structure, and plant species, and even different biomass distribution patterns. Consequently, their responses to grazing exclusion vary widely. In this study, we found that grassland type significantly influenced the effect of GE on soil microbial alpha diversity (Zhang et al., 2023), indicating that different management strategies should be adopted for the restoration of various degraded grassland type in practice. Both microbial Shannon and microbial richness in semidesert grasslands were higher than in alpine and temperate grasslands, perhaps because semidesert grasslands have relatively harsh environments (e.g., lower plant diversity and biomass, lower soil fertility, and poor structure) (Cao et al., 2024). Previous studies have demonstrated that GE had a stronger positive effect on plant biomass and SOC accumulation in desert grasslands than in other grassland types (Xiong et al., 2016; Yang et al., 2021). Similarly, our results also observed that GE had a greater effect on plant Shannon index, SOC, total nitrogen (TN), and available nitrogen (AN) in semidesert grassland compared to alpine and temperate grasslands (**Supplementary Figures S2**, **S3**). Moreover, this discrepancy indicates that the benefits of grazing exclusion in promoting microbial diversity may be more pronounced in resource-limited environments.

Numerous studies have confirmed that GE duration can obviously change plant communities, soil nutrient dynamics, and microbial communities. Our meta-analysis found that soil microbial diversity significantly increased after long-term GE. A possible interpretation is that soils under long-term GE have a better structure and accumulate more nutrients (Sun et al., 2020), as supported by our findings of long-term GE enhancing SOC, TN, AN, and MBC (**Supplementary Figure S3**). Furthermore, we found that mean annual temperature (MAT) was a key mediator of the variability in the soil microbial richness response to grazing exclusion. High MAT can increase nutrient accessibility to soil organisms by enhancing the decomposition rate of plant residues, consequently increasing microbial richness (Liu et al., 2021; Shu et al., 2022). The response of microbial diversity to grazing exclusion was not significantly affected by soil depth, suggesting that the impact of grazing exclusion on microbial communities remained consistent across different soil depths. This could be attributed to the adaptability of microbes to environmental changes or may reflect the influence of other factors within the grassland ecosystem on microbial communities.

Additionally, grazing intensity and soil type may be important factors affecting the influence of GE on soil microbial diversity. Unfortunately, this information is not included in our dataset due to data limitations. Therefore, this knowledge gap should be analyzed in future research.

### 4.3 Positive relationship between microbial diversity and EMF

Previous meta-analyses have shown that GE can enhance plant biomass, SOC accumulation, and soil fertility (Gao and Carmel, 2020; Sun et al., 2020; Qu et al., 2024). Our meta-analysis also found that GE increased plant belowground biomass, SOC, nutrient availability, soil enzyme activities, and even the EMF index. Notably, GE decreased the EMF index and some individual functions in humid regions (**Supplementary Figure S3**), indicating that GE is not be an effective approach to improve ecosystem multifunctionality in humid regions.

Grazing exclusion can alter ecosystem functioning by changing biodiversity, as biodiversity is tightly linked to ecosystem functioning in grasslands (Liu et al., 2022). Using the EMF index to evaluate GE effects, our results observed positive relationships between the EMF index and both plant Shannon and microbial Shannon, supporting our hypothesis. This suggests that both above- and belowground biodiversity play important roles in supporting ecosystem functions. Indeed, grasslands with high biodiversity are generally more productive and efficient in resource use (Navrátilová et al., 2018). Moreover, grasslands with high biodiversity can maximize rates of nutrient cycling (Oelmann et al., 2007; Shu et al., 2022). Interestingly, we observed that the correlation coefficient between the EMF index and plant Shannon is higher than its correlation with microbial Shannon. This finding supports a recent study showing that plant diversity has a stronger impact on EMF compared to microbial diversity under GE (Zhang et al., 2023). In addition, we found that grassland type determines the relationship between EMF and biodiversity. We observed that plant Shannon was only positively correlated with EMF in alpine grassland, while microbial Shannon was only positively correlated with EMF in temperate grassland (**Supplementary Table S3**). These findings imply that the relative importance of plant and soil microbial diversity in regulating the influence of GE on EMF differed. Overall, our meta-analysis suggests the fundamental importance of restoring above- and belowground biodiversity for improving EMF under GE.

### 4.4 Limitation and implications for grassland management

Our meta-analysis indicated that GE had an overall positive effect on soil microbial diversity and its functionality in grassland ecosystems. However, this impact was contingent upon factors including climatic region, grassland type, and GE duration. This implies an interplay between direct GE effects and indirect environmental changes, emphasizing comprehensive consideration of these factors during GE implementation. Given the negative influences of GE on soil microbial diversity and EMF in humid regions, GE may not improved microbial diversity and ecosystem functions in humid regions. It is worth noting that there was unavoidable shortcoming in our meta-analysis. For instance, the observation of most individual ecosystem functions is very limited. Additionally, insufficient information prevented assessment of crucial factors like initial soil and plant conditions, historical grazing processes, and pasture types.

## 5 Conclusion

Our meta-analysis evaluated the influences of grazing exclusion on soil microbial diversity and its functionality based on field studies. We found that GE significantly increased soil microbial diversity and EMF. However, the performance of microbial Shannon and EMF index varied substantially depending on climatic regions, grassland types and grazing exclusion duration. Specifically, grazing exclusion had a stronger positive effect on microbial diversity in semi-desert grasslands than in temperate and alpine grasslands. Furthermore, grazing exclusion may not be an effective approach to conserve microbial diversity and improve ecosystem multifunctionality in humid regions. Moreover, our study provides evidence that ecosystem multifunctionality is significantly and positively related to both microbial diversity and plant diversity at a large scale.

## Data availability statement

The raw data supporting the conclusions of this article will be made available by the authors, without undue reservation.

## Author contributions

XS: Funding acquisition, Methodology, Supervision, Writing – original draft, Validation, Visualization. QY: Funding acquisition, Methodology, Visualization, Writing – original draft, Writing – review & editing. HH: Data curation, Methodology, Writing – review & editing. LX: Writing – review & editing. HT: Investigation, Writing – review & editing. XL: Writing – review & editing, Funding acquisition, Methodology, Visualization, Writing – original draft. JW: Writing – review & editing. YL: Writing – review & editing. YZ: Writing – review & editing. LD: Writing – review & editing. WL: Funding acquisition, Writing – review & editing.
