# Supplementary material for: Effects of grazing exclusion on soil microbial diversity and its functionality in grasslands: a meta-analysis
Source: Front Plant Sci. 2024 Mar 19;15:1366821. doi: 10.3389/fpls.2024.1366821 (PMC10985342; doi:10.3389/fpls.2024.1366821)
Supplement: Supplementary file 1 [file Image_1.pdf]

Supplementary material for

Effects of grazing exclusion on soil microbial diversity and its functionality in grasslands：A meta-analysis

Xiangyang Shu [a, †, *], Qinxin Ye [b, †], Han Huang [c, †], Longlong Xia [d], Hao Tang [a], Xinyi Liu [b], Jianwei Wu [b], Yiding Li [e], Yanyan Zhang [e], Liangji Deng [e], Weijia Liu [b, *]

[a] *Key Laboratory of Land Resources Evaluation and Monitoring in Southwest, Ministry of Education, Sichuan Normal University, Chengdu, 610068, China*

[b] *Institute of Agricultural Bioenvironment and Energy, Chengdu Academy of Agriculture and Forestry Sciences, Chengdu 611130, China*

[c] *College of Economics and Management, Xinjiang Agricultural University, Urumqi 830052, China*

[d] *State Key Laboratory of Soil and Sustainable Agriculture, Institute of Soil Science, Chinese Academy of Sciences, Nanjing 82467, China*

[e] *College of Resources, Sichuan Agricultural University, Chengdu, 611130, China*

[*]Corresponding author: Xiangyang Shu and Weijia Liu

E-mail Address: xyshu@sicnu.edu.cn (X. Shu) and Liuweijia27@163.com (W. Liu).

Address: Key Laboratory of Land Resources Evaluation and Monitoring in Southwest, Ministry of Education, Sichuan Normal University, Jingan Road, Chengdu 610066, China.

Phone: +086-182-0280-9282

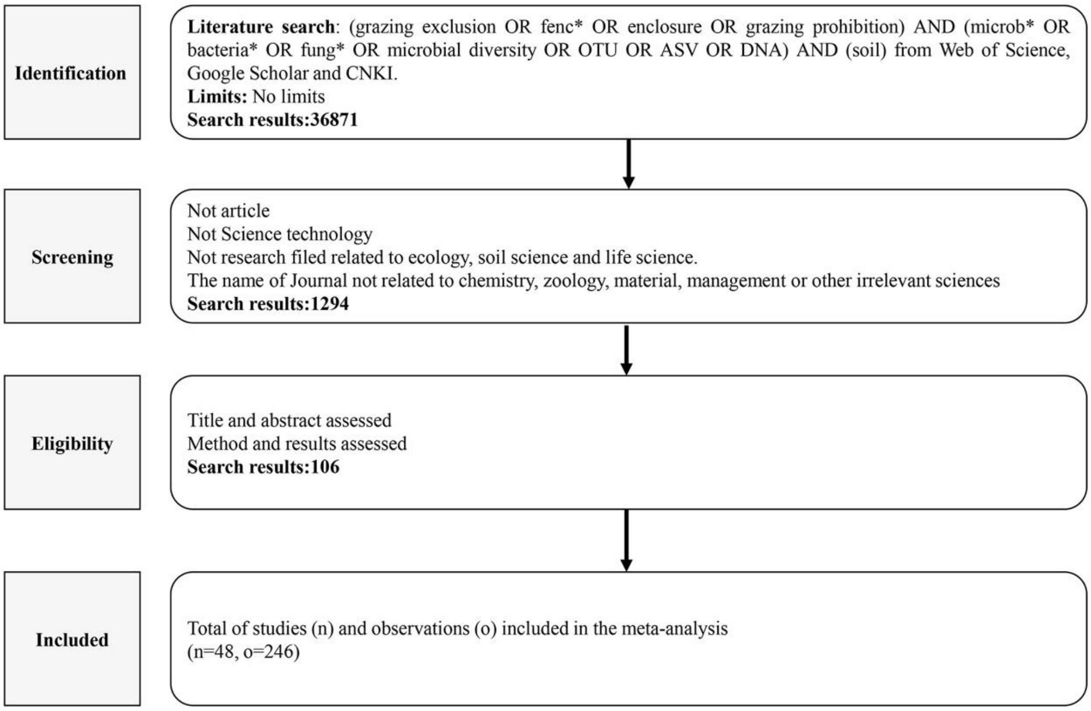

Supplementary Figure S1 Article selection process using Preferred Reporting Items for Systematic Reviews (PRISMA) guidelines.

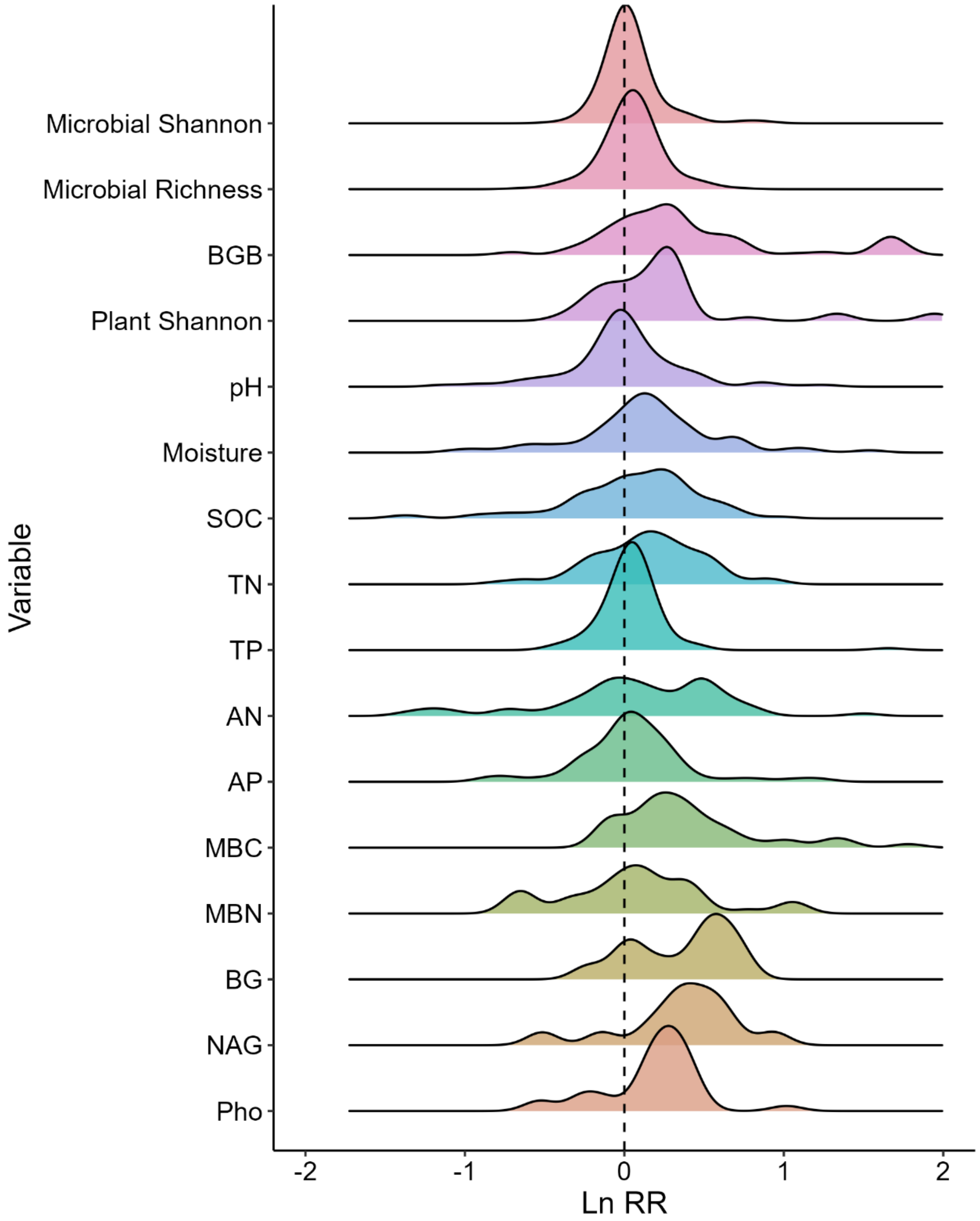

Supplementary Figure S2 Density distributions of response ratios (LnRR) of plant, soil and microbial variables. BGB, belowground biomass; SOC, soil organic carbon; TN, total nitrogen; TP, total phosphorus; AN, available nitrogen; AP, available phosphorus; MBC, microbial biomass carbon; MBN, microbial biomass nitrogen; BG, β-glucosidase; NAG, β-1,4,-N-acetyl-glucosaminnidase; Pho, phosphatase.

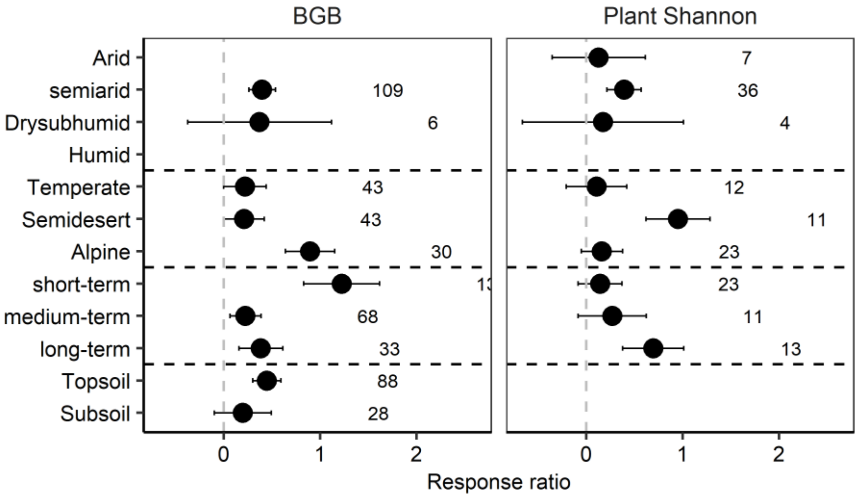

Supplementary Figure S3 Response of below-ground biomass and plant Shannon index to grazing exclusion across different climate, grassland type, and grazing duration. BGB, belowground biomass.

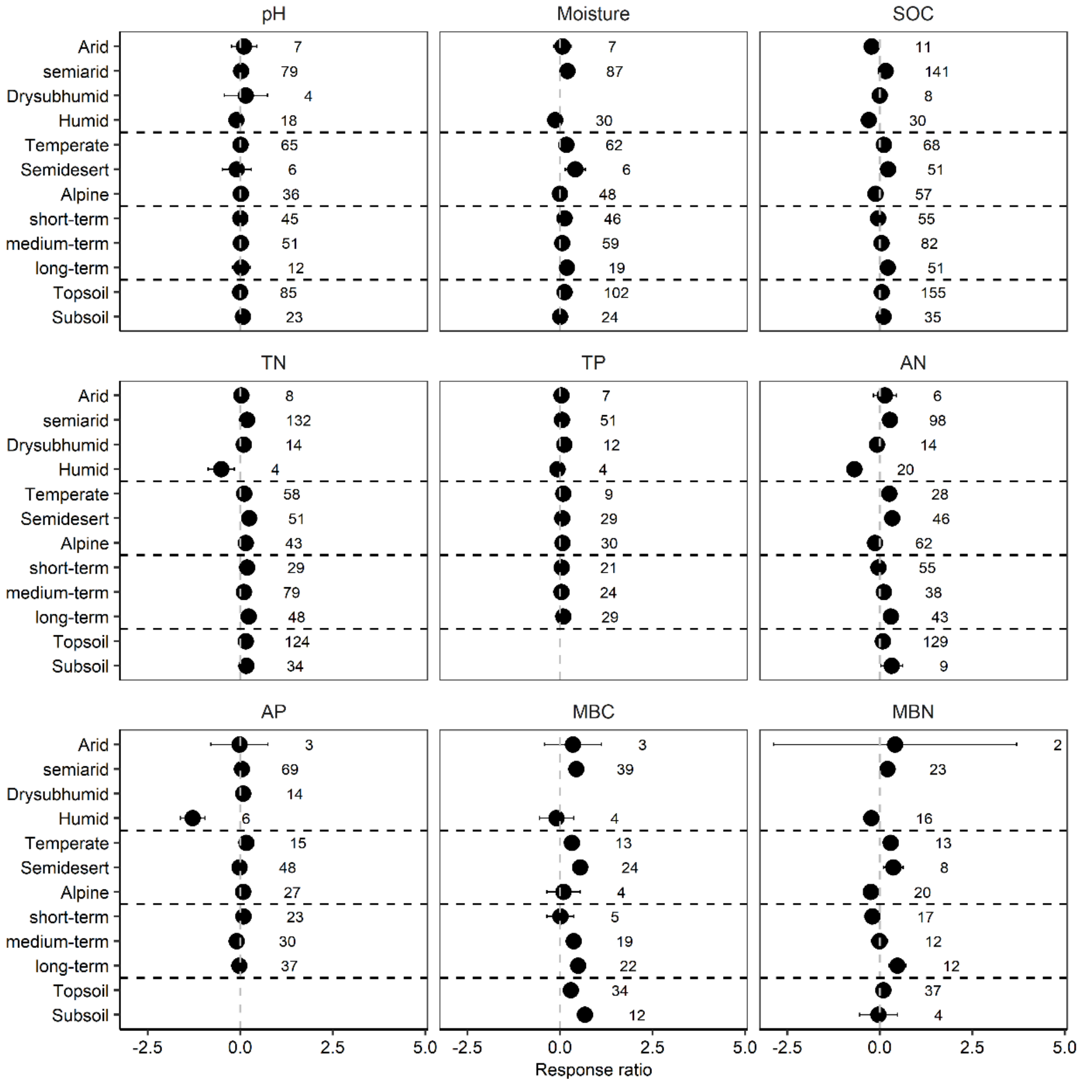

Supplementary Figure S4 Responses of soil variables to grazing exclusion across different climate zones, grassland types, grazing exclusion, and soil depths. SOC, soil organic carbon; TN, total nitrogen; TP, total phosphorus; AN, available nitrogen; AP, available phosphorus; MBC, microbial biomass carbon; MBN, microbial biomass nitrogen; BG, β-glucosidase; NAG, β-1,4,-N-acetyl-glucosaminnidase; Pho, phosphatase.

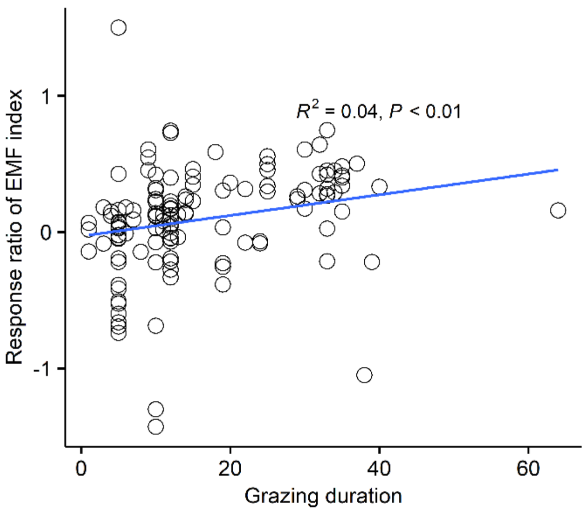

Supplementary Figure S5 Relationship between grazing duration and response ratio of ecosystem multifunctionality (EMF) index.
