# Supplementary material for: Effects of grazing exclusion on soil microbial diversity and its functionality in grasslands: a meta-analysis
Source: Front Plant Sci. 2024 Mar 19;15:1366821. doi: 10.3389/fpls.2024.1366821 (PMC10985342; doi:10.3389/fpls.2024.1366821)
Supplement: Supplementary file 2 [file Table_1.pdf]

Supplementary material for

Effects of grazing exclusion on soil microbial diversity and its functionality in grasslands：A meta-analysis

Xiangyang Shu [a, †, *], Qinxin Ye [b, †], Han Huang [c, †], Longlong Xia [d], Hao Tang [a], Xinyi Liu [b], Jianwei Wu [b], Yiding Li [e], Yanyan Zhang [e], Liangji Deng [e], Weijia Liu [b, *]

[a] *Key Laboratory of Land Resources Evaluation and Monitoring in Southwest, Ministry of Education, Sichuan Normal University, Chengdu, 610068, China*

[b] *Institute of Agricultural Bioenvironment and Energy, Chengdu Academy of Agriculture and Forestry Sciences, Chengdu 611130, China*

[c] *College of Economics and Management, Xinjiang Agricultural University, Urumqi 830052, China*

[d] *State Key Laboratory of Soil and Sustainable Agriculture, Institute of Soil Science, Chinese Academy of Sciences, Nanjing 82467, China*

[e] *College of Resources, Sichuan Agricultural University, Chengdu, 611130, China*

[*]Corresponding author: Xiangyang Shu and Weijia Liu

E-mail Address: xyshu@sicnu.edu.cn (X. Shu) and Liuweijia27@163.com (W. Liu).

Address: Key Laboratory of Land Resources Evaluation and Monitoring in Southwest, Ministry of Education, Sichuan Normal University, Jingan Road, Chengdu 610066, China.

Phone: +086-182-0280-9282

Supplementary Table S1 Results of Egger's tests for microbial diversity and ecosystem multifunctionality (P_E < 0.05 marked in red, suggesting a significant publication bias).

Variable	P_E value
Shannon	0.28168
Richness	0.89163
EMF	0.07924

Supplementary Table S2 Relationships between microbial diversity and plant variables, soil variables and ecosystem multifunctionality index across different microbial groups. Asterisks denote significance:* P<0.05, ** P<0.01, *** P<0.001.

variables	Bacteria		Fungi	
	Shannon	Richness	Shannon	Richness
PS	0.3	-0.12	-0.22	-0.49
BGB	-0.072	0.16	0.13	0.35
pH	-0.001	0.33**	0.14	0.47**
Moisture	0.055	0.15	0.18	0.055
SOC	0.27**	0.27	0.23	0.17
TN	0.3**	0.23	0.16	-0.24
TP	0.3	0.43**	-0.079	0.13
AN	0.24*	0.33**	0.31*	0.11
AP	0.33*	0.46***	-0.23	0.082
MBC	-0.073	0.45*	-0.21	0.22
MBN	0.27	0.44	0.018	0.24
BG	-0.17	-0.22	0.3	-0.5
NAG	0.011	-0.13	0.3	-0.5
Pho	0.24	-0.055	-0.21	-0.57
EMF index	0.23*	0.32**	0.19	0.06

Supplementary Table S3 Relationships between plant Shannon, microbial Shannon, and microbial richness and ecosystem multifunctionality index across different grassland types. Asterisks denote significance:* *P*<0.05, ** *P*<0.01.

	Alpine	Temperate	Semidesert
Plant Shannon	0.42*	0.46	0.31
Microbial Shannon	0.097	0.35**	-0.31
Microbial richness	0.088	0.22	0.096

References List

[1] Wang, L., Delgado‐Baquerizo, M., Zhao, X., Zhang, M., Song, Y., Cai, J., ... & Xin, X. (2020). Livestock overgrazing disrupts the positive associations between soil biodiversity and nitrogen availability. *Functional Ecology*, *34*(8), 1713-1720.

[2] Yao, M., Rui, J., Li, J., Wang, J., Cao, W., & Li, X. (2018). Soil bacterial community shifts driven by restoration time and steppe types in the degraded steppe of Inner Mongolia. *Catena*, *165*, 228-236.

[3] Chen, L., Shi, J., Bao, Z., & Baoyin, T. (2020). Soil fungal networks are more sensitive to grazing exclusion than bacterial networks. *PeerJ*, *8*, e9986.

[4] Zhou, J., Zhang, M., Raza, S. T., Yang, S., Liu, J., Cai, M., ... & Wu, J. (2023). Fungal but not bacterial β-diversity decreased after 38-year-long grazing in a southern grassland. *Plant and Soil*, 1-13.

[5] Wang, Y. H., Tian, L. M., Ai, Y., Chen, S. Y., & Mipam, T. D. K. (2022). Effects of short-term yak grazing intensity on soil bacterial communities in an alpine meadow of the Northwest Sichuan Plateau. *Acta Ecol. Sin*, *42*(4), 1549-1559.

[6] JIANG, A. J., DONG, Y. Q., ASITAIKEN, J., ZHOU, S. J., NIE, T. T., LI, C. Y., ... & AN, S. Z. Effects of Grazing Exclusion on Soil Bacterial Community Characteristics in different Grassland Types. *Acta Agrestia Sinica*, *30*(10), 2600.

[7] Zhang, C., Li, J., Wang, J., Liu, G., Wang, G., Guo, L., & Peng, S. (2019). Decreased temporary turnover of bacterial communities along soil depth gradient during a 35-year grazing exclusion period in a semiarid grassland. *Geoderma*, *351*, 49-58.

[8] Su, J., Ji, W., Sun, X., Wang, H., Kang, Y., & Yao, B. (2023). Effects of different management practices on soil microbial community structure and function in alpine grassland. *Journal of Environmental Management*, *327*, 116859.

[9] Li, N., Chang, R., Jiang, H., Tariq, A., Sardans, J., Peñuelas, J., ... & Zhou, X. (2022). Combined livestock grazing-exclusion and global warming decreases nitrogen mineralization by changing soil microbial community in a Tibetan alpine meadow. *Catena*, *219*, 106589.

[10] Li, Y., Dong, S., Gao, Q., Fan, C., Fayiah, M., Ganjurjav, H., ... & Li, S. (2022). Grazing changed plant community composition and reduced stochasticity of soil microbial community assembly of alpine grasslands on the Qinghai-Tibetan Plateau. *Frontiers in Plant Science*, *13*, 864085.

[11] Wang, Q., Bao, Y., Liu, X., & Du, G. (2014). Spatio-temporal dynamics of arbuscular mycorrhizal fungi associated with glomalin-related soil protein and soil enzymes in different managed semiarid steppes. *Mycorrhiza*, *24*, 525-538.

[12] Wang, Z., Zhang, Q., Staley, C., Gao, H., Ishii, S., Wei, X., ... & Sadowsky, M. J. (2019). Impact of long-term grazing exclusion on soil microbial community composition and nutrient availability. *Biology and Fertility of Soils*, *55*, 121-134.

[13] Yang, P. N., Li, X. L., Li, C. Y., & Duan, C. W. (2023). Response of Soil Microbial Diversity to Long-term Enclosure in Degraded Patches of Alpine Meadow in the Source Zone of the Yellow River. *Huan Jing ke Xue*, *44*(4), 2293-2303.

[14] Guo, Q., Wen, Z., Ghanizadeh, H., Fan, Y., Zheng, C., Yang, X., ... & Li, W. (2023). Stochastic processes dominate assembly of soil fungal community in grazing excluded grasslands in northwestern China. *Journal of Soils and Sediments*, *23*(1), 156-171.

[15] Zhang, R., Wang, Z., Niu, S., Tian, D., Wu, Q., Gao, X., ... & Han, G. (2021). Diversity of plant and soil microbes mediates the response of ecosystem multifunctionality to grazing disturbance. *Science of the total environment*, *776*, 145730.

[16] Fernández-Guisuraga, J. M., Calvo, L., Ansola, G., Pinto, R., & de Miera, L. E. S. (2022). The effect of sheep grazing abandonment on soil bacterial communities in productive mountain grasslands. *Science of The Total Environment*, *851*, 158398.

[17] Jingjing, Y., Huiqin, G., Fry, E. L., Jonathan, R., Shiming, T., Ting, Y., & Weibo, R. (2022). Plant roots send metabolic signals to microbes in response to long-term overgrazing. *Science of The Total Environment*, *842*, 156241.

[18] Ma, C. H., Hao, X. H., He, F. C., Baoyin, T. G., Yang, J. J., & Dong, S. K. (2022). Effects of seasonal grazing on plant and soil microbial diversity of typical temperate grassland. *Frontiers in Plant Science*, *13*, 1040377.

[19] Wang, Z., Li, X., Ji, B., Struik, P. C., Jin, K., & Tang, S. (2021). Coupling between the responses of plants, soil, and microorganisms following grazing exclusion in an overgrazed grassland. *Frontiers in Plant Science*, *12*, 640789.

[20] Peng, W. E. I., Shazhou, A. N., Mei, K. E., Chao, L., Yurong, H., Jiyong, L., ... & Junpeng, J. (2021). Effects of enclosure on

plant and soil restoration in the junggar desert. *Journal of Resources and Ecology*, *12*(6), 840-848.

[21] Fan, D., Ji, M., Wu, J., Chen, H., Jia, H., Zhang, X., ... & Kong, W. (2023). Grazing does not influence soil arbuscular mycorrhizal fungal diversity, but increases their interaction complexity with plants in dry grasslands on the Tibetan Plateau. *Ecological Indicators*, *148*, 110065.

[22] Wu, Y., Chen, D., Delgado-Baquerizo, M., Liu, S., Wang, B., Wu, J., ... & Bai, Y. (2022). Long-term regional evidence of the effects of livestock grazing on soil microbial community structure and functions in surface and deep soil layers. *Soil Biology and Biochemistry*, *168*, 108629.

[23] Farrell, H. L., Barberán, A., Danielson, R. E., Fehmi, J. S., & Gornish, E. S. (2020). Disturbance is more important than seeding or grazing in determining soil microbial communities in a semiarid grassland. *Restoration Ecology*, *28*, S335-S343.

[24] Wang, Z., Jiang, S., Struik, P. C., Wang, H., Jin, K., Wu, R., ... & Ta, N. (2023). Plant and soil responses to grazing intensity drive changes in the soil microbiome in a desert steppe. *Plant and Soil*, *491*(1), 219-237.

[25] Zheng, J., Zhang, B., Zhang, F., Zhao, T., Wang, Q., Han, G., & Zhao, M. (2023). Effects of fencing on near-term ecosystem multifunctionality in a typical steppe in Inner Mongolia. *Agriculture, Ecosystems & Environment*, *342*, 108238.

[26] Zhang, L., Wang, J., Wang, X. T., Liao, L. R., Wan, Q., Liu, G. B., & Zhang, C. (2021). Effect of restoration types on the community structure of microbes harboring nifH and chiA genes in alpine meadow. *The Journal of Applied Ecology*, *32*(12), 4349-4358. （In Chinese）

[27] Sun, J., Wang, P., Wang, H., & Yu, X. (2021). Changes in plant communities, soil characteristics, and microbial communities in alpine meadows degraded to different degrees by pika on the Qinghai–Tibetan Plateau. *Global Ecology and Conservation*, *27*, e01621.

[28] Yin, Y., Wang, Y., Li, S., Liu, Y., Zhao, W., Ma, Y., & Bao, G. (2021). Soil microbial character response to plant community variation after grazing prohibition for 10 years in a Qinghai-Tibetan alpine meadow. *Plant and Soil*, *458*, 175-189.

[29] Wang, H., Li, J., Zhang, Q., Liu, J., Yi, B., Li, Y., ... & Di, H. (2019). Grazing and enclosure alter the vertical distribution of organic nitrogen pools and bacterial communities in semiarid grassland soils. *Plant and Soil*, *439*, 525-539.

[30] Ya-li, Y. I. N., Yu-qin, W. A. N. G., Shi-xiong, L. I., Yan, L. I. U., Wen, Z. H. A. O., Yu-shou, M. A., & Gen-sheng, B. A. O. (2019). Effects of enclosing on soil microbial community diversity and soil stoichiometric characteristics in a degraded alpine meadow. *Yingyong Shengtai Xuebao*, *30*(1).

[31] Zhang, Y., Gao, X., Hao, X., Alexander, T. W., Shi, X., Jin, L., & Thomas, B. W. (2020). Heavy grazing over 64 years reduced soil bacterial diversity in the foothills of the Rocky Mountains, Canada. *Applied Soil Ecology*, *147*, 103361.

[32] Aldezabal, A., Moragues, L., Odriozola, I., & Mijangos, I. (2015). Impact of grazing abandonment on plant and soil microbial communities in an Atlantic mountain grassland. *Applied Soil Ecology*, *96*, 251-260.

[33] Wang, Z., Ding, Y., Jin, K., Struik, P. C., Sun, S., Ji, B., ... & Li, X. (2022). Soil bacterial and fungal communities are linked with plant functional types and soil properties under different grazing intensities. *European Journal of Soil Science*, *73*(1), e13195.

[34] Aldezabal, A., Moragues, L., Odriozola, I., & Mijangos, I. (2015). Impact of grazing abandonment on plant and soil microbial communities in an Atlantic mountain grassland. *Applied Soil Ecology*, *96*, 251-260.

[35] Zhang, C., Liu, G., Song, Z., Wang, J., & Guo, L. (2018). Interactions of soil bacteria and fungi with plants during long-term grazing exclusion in semiarid grasslands. *Soil Biology and Biochemistry*, *124*, 47-58.

[36] Zhang, L., Wang, X., Wang, J., Wan, Q., Liao, L., Liu, G., & Zhang, C. (2021). Grazing exclusion reduces soil N_2O emissions by regulating nirK-and nosZ-type denitrifiers in alpine meadows. *Journal of Soils and Sediments*, *21*, 3753-3769.

[37] Wang, J., Li, W., Cao, W., Abalori, T. A., Liu, Y., Xin, Y., ... & Zhang, D. (2021). Soil bacterial community responses to short-term grazing exclusion in a degraded alpine shrubland–grassland ecotone. *Ecological Indicators*, *130*, 108043.

[38] Cao, J., Jiao, Y., Che, R., Holden, N. M., Zhang, X., Biswas, A., & Feng, Q. (2022). The effects of grazer exclosure duration on soil microbial communities on the Qinghai-Tibetan Plateau. *Science of The Total Environment*, *839*, 156238.

[39] Wang, J., Wang, X., Liu, G., Wang, G., & Zhang, C. (2021). Grazing-to-fencing conversion affects soil microbial composition,

functional profiles by altering plant functional groups in a Tibetan alpine meadow. *Applied Soil Ecology*, *166*, 104008.

[40] Rong, Y., Monaco, T. A., Liu, Z., Zhao, M., & Han, G. (2022). Soil microbial community structure is unaltered by grazing intensity and plant species richness in a temperate grassland steppe in northern China. *European Journal of Soil Biology*, *110*, 103404.

[41] Qin, Y., Xiaofang, Z., Adamowski, J. F., Biswas, A., Holden, N. M., & Hu, Z. (2021). Grassland grazing management altered soil properties and microbial β-diversity but not α-diversity on the Qinghai-Tibetan Plateau. *Applied Soil Ecology*, *167*, 104032.

[42] Cheng, J., Jing, G., Wei, L., & Jing, Z. (2016). Long-term grazing exclusion effects on vegetation characteristics, soil properties and bacterial communities in the semi-arid grasslands of China. *Ecological Engineering*, *97*, 170-178.

[43] Wang, J., Wang, X., Liu, G., Wang, G., & Zhang, C. (2021). Grazing-to-fencing conversion affects soil microbial composition, functional profiles by altering plant functional groups in a Tibetan alpine meadow. *Applied Soil Ecology*, *166*, 104008.

[44] Fan, J., Zhang, C., Jin, H., Zhang, J., & Han, G. (2021). Grazing accelerates labile and recalcitrant soil carbon loss driving by rare microbial taxa in a desert steppe. *Land Degradation & Development*, *32*(15), 4241-4253.

[45] de Araujo Pereira, A. P., Lima, L. A. L., Bezerra, W. M., Pereira, M. L., Normando, L. R. O., Mendes, L. W., ... & Melo, V. M. M. (2021). Grazing exclusion regulates bacterial community in highly degraded semiarid soils from the Brazilian Caatinga biome. *Land Degradation & Development*, *32*(6), 2210-2225.

[46] Wang, Z., Deng, H., Li, F., Sun, Y., & Hong, S. (2023). Optimized soil bacterial structure following grazing exclusion promotes soil nutrient cycling and plant growth. *Journal of Arid Environments*, *213*, 104977.

[47] Zhu Y, Wu Y B, An Y T. Effects of grazing prohibition on soil microbial community structure based on high-throughput sequencing. Acta Ecologica Sinica, 2022, 42(17): 7137-7146. (In Chinese)

[48] Yang, P. N., Li, X. L., Li, C. Y., & Duan, C. W. (2023). Response of Soil Microbial Diversity to Long-term Enclosure in Degraded Patches of Alpine Meadow in the Source Zone of the Yellow River. *Environmental Science*, *44*(4), 2293-2303. (In Chinese)
